# Dew point temperature affects ascospore release of allergenic genus *Leptosphaeria*

**DOI:** 10.1007/s00484-018-1500-z

**Published:** 2018-01-27

**Authors:** Magdalena Sadyś, Joanna Kaczmarek, Agnieszka Grinn-Gofron, Victoria Rodinkova, Alex Prikhodko, Elena Bilous, Agnieszka Strzelczak, Robert J. Herbert, Malgorzata Jedryczka

**Affiliations:** 1 Rothamsted Research, West Common, Harpenden, AL5 2JQ UK; 2 Institute of Science and the Environment, University of Worcester, Henwick Grove, Worcester, WR2 6AJ UK; 3 Institute of Plant Genetics, Polish Academy of Sciences, Strzeszynska 34, 60-479 Poznan, Poland; 4 Department of Plant Taxonomy and Phytogeography, Faculty of Biology, University of Szczecin, Waska 13, 71-415 Szczecin, Poland; 5 National Pirogov Memorial Medical University, 56 Pirogov str., 21018 Vinnytsya, Ukraine; 6 Zaporizhia State Medical University, 26 Maiakovskij str., 69035 Zaporizhia, Ukraine; 7 Faculty of Food Sciences and Fisheries, Department of Food Process Engineering, West Pomeranian University of Technology, Papieza Pawla VI 3, 71-459 Szczecin, Poland

**Keywords:** Species-environment relationship, Disease forecasting, Bio-climate, Dew point temperature, Multivariate regression trees, Artificial neural networks

## Abstract

The genus *Leptosphaeria* contains numerous fungi that cause the symptoms of asthma and also parasitize wild and crop plants. In search of a robust and universal forecast model, the ascospore concentration in air was measured and weather data recorded from 1 March to 31 October between 2006 and 2012. The experiment was conducted in three European countries of the temperate climate, i.e., Ukraine, Poland, and the UK. Out of over 150 forecast models produced using artificial neural networks (ANNs) and multivariate regression trees (MRTs), we selected the best model for each site, as well as for joint two-site combinations. The performance of all computed models was tested against records from 1 year which had not been used for model construction. The statistical analysis of the fungal spore data was supported by a comprehensive study of both climate and land cover within a 30-km radius from the air sampler location. High-performance forecasting models were obtained for individual sites, showing that the local micro-climate plays a decisive role in biology of the fungi. Based on the previous epidemiological studies, we hypothesized that dew point temperature (DPT) would be a critical factor in the models. The impact of DPT was confirmed only by one of the final best neural models, but the MRT analyses, similarly to the Spearman’s rank test, indicated the importance of DPT in all but one of the studied cases and in half of them ranked it as a fundamental factor. This work applies artificial neural modeling to predict the *Leptosphaeria* airborne spore concentration in urban areas for the first time.

**Electronic supplementary material:**

The online version of this article (10.1007/s00484-018-1500-z) contains supplementary material, which is available to authorized users.

## Introduction

Pollen and molds are the most commonly identified and described aeroallergens which constitute aeroplankton and may cause respiratory problems in immune sensitive individuals. Among molds, the best known allergens are in the genera *Alternaria*, *Cladosporium*, *Aspergillus*, *Penicillium* and to a lesser extent *Ganoderma* (O’Connor et al. 2014; Jedryczka et al. 2015). Thus far, no research on potential allergens has been conducted in other types of fungi. However, many medical papers suggest that additional fungal species may contribute to allergies and asthma (see, for example, Tilak 1991; Green et al. 2005).

Fungi of the genus *Leptosphaeria* are well-known plant pathogens (see, for example, West et al. 2001; Fitt et al. 2006), and there is recent evidence that ascospores of *Leptosphaeria* species can also contribute to the symptoms of inhalatory allergies (Jedryczka et al. 2016). Authors found that *Leptosphaeria* spp. produce allergenic proteins highly similar to those commonly known in the genera *Alternaria*, *Cladosporium*, *Curvularia*, *Penicillium*, and *Aspergillus* and concluded that *Leptosphaeria* spp. spores played an important role in autumn asthma (Jedryczka et al. 2016).

The release and subsequent spread of spores is influenced by many environmental factors. Release of spores depends, among other factors, on the type of release mechanism. The fruiting bodies of the ascomycetes protect the spores and asci during dry periods, preparing them for release during times of high moisture; the most common source of which is dew and rain (Ingold 1985). In Ascomycota, the division of fungi containing the genus *Leptosphaeria*, several mechanisms for the opening of asci and liberation of ascospores have been described (Ingold 1985; Trail 2007). The *Leptosphaeria* genus has an ostiolate ascocarp type. When the ascus becomes turgid, the outer wall ruptures at the apex and extends considerably, at least two or three times its length. The endoascus comes out of the ostiole and explodes to release the spores (Deacon 2003).

*Leptosphaeria* spp. are common pathogens of brassica crops, including oilseed rape and, owing to the prevalence of this crop, they are often present in air samples (Huang et al. 2005; Jedryczka et al. 2016). When sexual reproduction of *Leptosphaeria* spp. takes place, fruiting bodies (pseudothecia) are formed on the stubble of oilseed rape originating from previous growing seasons (West and Fitt 2005), with the spores released from the most recent (Hall 1992), as well as previous crops (Khangura et al. 2007). The release of ascospores requires the production of fully developed pseudothecia (Kaczmarek and Jedryczka 2011). The maturation rate of the fruiting bodies of *Leptosphaeria* spp. (hereafter *Leptosphaeria*) and subsequent spore release depend on weather conditions (Savage et al. 2012), mainly moisture and air temperature (Salam et al. 2003; Huang et al. 2005). Studies by Toscano-Underwood et al. (2003) indicated that the influence of moisture (rain or heavy dew) is more crucial than air temperature. Therefore, it could be hypothesized that dew point temperature is a good parameter for forecasting the occurrence of *Leptosphaeria* spores in the air from nearby affected crops.

The patterns of ascospore release and weather data differ between countries (Huang et al. 2005; Lob et al. 2013) and sites under study (Oliveira et al. 2009). Therefore, it is highly desirable to find out which meteorological factors would most accurately allow forecast of the timing of appearance of this phytopathogens and aeroallergens in a particular season and region. Artificial neural modeling is a method which can predict further values based on an analysis of data series (Tadeusiewicz and Lula 2001). Neural networks have already been successfully applied in the analysis and forecasting of allergenic fungal spores, such as *Alternaria* spp. (Grinn-Gofroń and Strzelczak 2008a; Astray et al. 2010; Kumar et al. 2013), *Cladosporium* spp. (Grinn-Gofroń and Strzelczak 2008b), and to some extent also for *Ganoderma* spp. (Kasprzyk et al. 2011; Sadyś et al. 2016). However, as this technique has not been yet used in modeling concentration of *Leptosphaeria* ascospores, the aim of this study was to use artificial neural network modeling for the first time in this genus. Moreover, as prediction of these spores could greatly help allergologists and plant pathologists in understanding ascospore outbreaks, an additional aim was to identify the weather parameters that allow the creation of robust and universal forecast models for *Leptosphaeria* ascospores. The studies were undertaken at four locations throughout Europe (Poland, the UK, and two sites in Ukraine), which substantially differ in weather conditions.

## Materials and methods

### Study sites

**Fig. 1:**
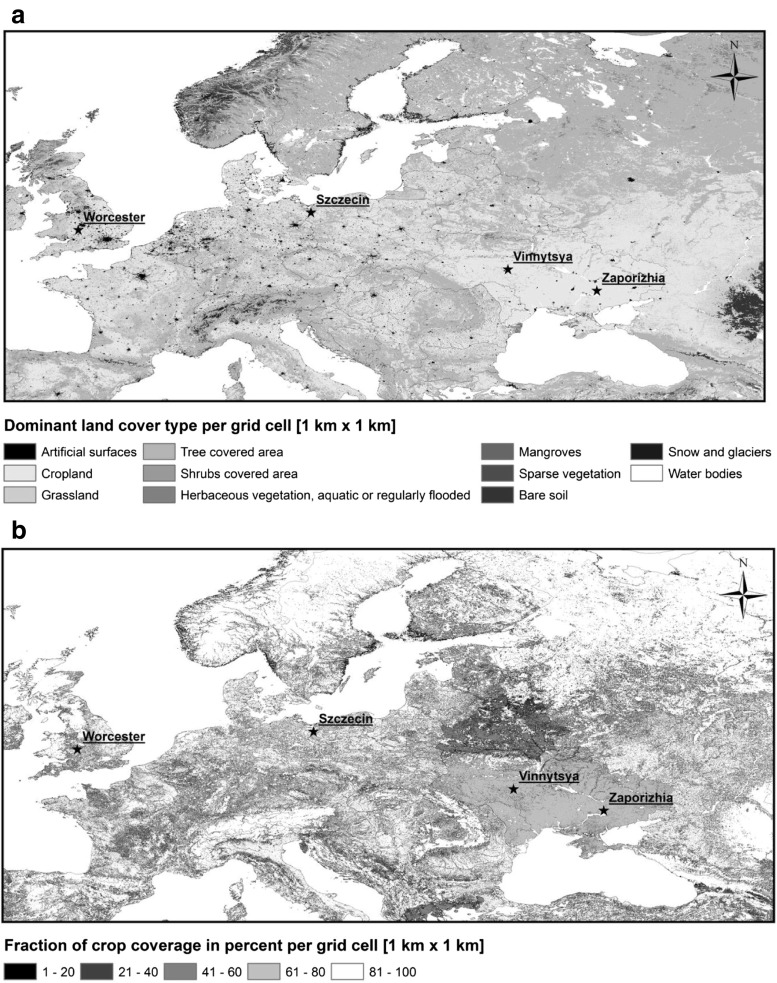
Distribution of land cover classes shows **a** the dominance of the agricultural areas in Europe, while **b** simultaneously the density between cultivated crops vary significantly between countries. Location of study sites in Poland, the UK, and Ukraine were marked with an asterisk

The concentration of airborne *Leptosphaeria* ascospores was measured for several consecutive years between 2006 and 2012 using four air samplers of the Hirst design (Hirst 1952). The spore traps were located on the rooftop of university buildings, i.e., University of Szczecin, Poland (53° 26’ N, 14° 32′ E), University of Worcester, UK (52° 11’ N, 2° 14’ W), National Medical University, Vinnytsya, Ukraine (49° 22’ N, 28° 44′ E), and Zaporizhia State Medical University, Ukraine (47° 83’ N, 35° 11’ W) at 21, 10, 25, and 20 m above ground level, respectively (Fig. 1) (Table 1).

**Table 1:** Characteristic of study sites, climate and weather data in Poland (PL), the United Kingdom (UK), and Ukraine (UA)

Site	Szczecin PL	Worcester UK	Vinnytsya UA	Zaporizhia UA
Geographic parameters	53° 26’ N 14° 32′ E	52° 11’ N 2° 13’ W	49° 14’ N 28° 29′ E	47° 50’ N 35° 10′ E
Location	North-west part of Poland	West Midlands in England	Central-west Ukraine	South-east Ukraine
% of forests	33	5	15	4
Other fractions	Non-irrigated arable land (31%), pastures (17%)	Non-irrigated arable land (41%), pastures (24%).	Farmlands (80%)	Farmlands (90%)
Climate	Temperate transitional	Temperate marine	Temperate continental	Moderate-continental
Annual mean temperature	9.6 ° C	9.5 °C	8.4 °C	5.1 °C
No. of days with snow cover	50–80	17	29–59	58
Sum of annual precipitation	550–700 mm	669 mm	594–638 mm	448 mm
Dominant wind	Western and south-western	South western	Western and south-western	South-western

### Ascospore sampling and identification

The method followed was as described by the British Aerobiology Federation (1995). Identification of *Leptosphaeria* spores was based on the morphological characteristics of the spores; ascospores are fusoid, ellipsoid, or cylindrical and their size is 18–120 μm × 4–15 μm (Dennis 1978). Color of the spores is yellowish or yellowish-brown to olivaceous and the cell wall surface is smooth. From two to several, cross septa may be present (Williams and Fitt, 1999), with one cell frequently enlarged (Dennis 1978). A typical ascospore of *Leptosphaeria* species contains six cells (Kaczmarek and Jedryczka 2011). Selected microscope slides were re-examined microscopically and tested for the presence of *Leptosphaeria* DNA, which confirmed accuracy of the data analysis (Jedryczka et al. 2016).

### Weather data

**Fig. 2:**
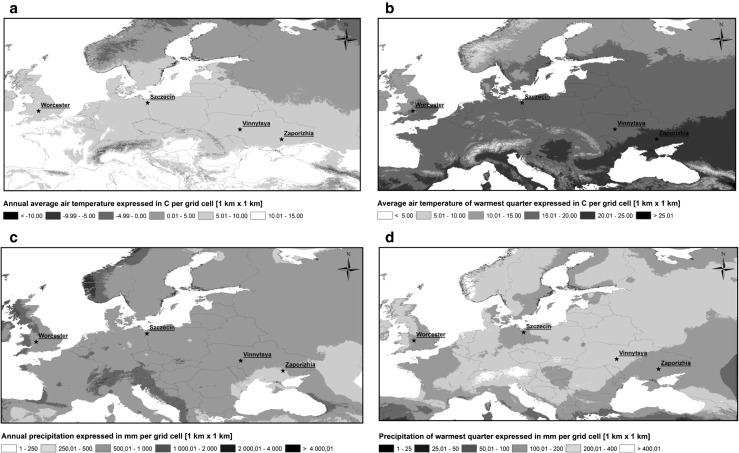
Selected bioclimatic indices were chosen: **a** annual mean temperature, **b** average air temperature of warmest quarter, **c** annual precipitation, **d** precipitation of warmest quarter. Location of study sites in Poland, the UK, and Ukraine were marked with an asterisk

The weather data were recorded using weather stations, which were co-located with spore traps. The weather parameters included in this study were mean air temperature, maximum air temperature, minimum air temperature, dew point temperature, relative humidity, precipitation, and mean wind speed. The maps of the selected bioclimatic indices in Europe with marked study sites are presented in Fig. 2.

### Statistical analyses

Since most of the typical statistical methods like multiple regression require normality of the variables and linearity of dependences between them, the first step was to check if the parameters followed normal distribution. Normality of variables was tested using the Kolmogorov-Smirnov, Lilliefors, and chi-square tests under *p* = 0.05. The next step was the assessment of linearity of relationships between spore abundance and meteorological factors with the help of scatter plots and the normality of residues from linear regression. Since neither normality nor linearity were fulfilled, the artificial neural networks (ANN) and multivariate regression trees (MRT) were applied. These methods were chosen because they do not require any assumptions about variables’ distribution or the form of relationships between them. They are applicable to data with high-order interactions, imbalance, and non-linear dependences. ANN and MRT models were created for data recorded in (a) Szczecin, Worcester, and jointly for Szczecin+Worcester using the data collected in 2006–2009 (1 Mar–31 Oct), (b) Vinnytsya, Zaporizhia, and jointly for Vinnytsya+Zaporizhia using the data collected in 2009–2012 (1 Mar–31 Oct). Two types of models were computed: (1) with dew point temperature and (2) without dew point temperature. We used 67% of data records to train the neural networks and 33% of the input to validate them. The quality of predictions of spore concentration based on meteorological factors was tested on data recorded at each site in 2009 (1 Mar–31 Oct).

For the sake of this study, multi-layer perceptrons (MLP) were applied since they mathematically perform a stochastic approximation of multivariate functions (Carling 1992; Lek and Guegan 1999; Osowski 1996). Consecutive neural network models were created and trained with the help of Automated Network Search (ANS), which tries a set of networks of various complexity and different activation functions (Statsoft 2011). Several thousand ANN models were tested. The number of hidden neurons ranged from 3 to 50 and seven different activation functions were used—linear, logistic, hyperbolic tangent, negative exponential, and sine. Finally, the top ten models were examined in each category. Neural networks were trained with the recommended technique, i.e., Broyden-Fletcher-Goldfarb-Shanno algorithm (BFGS), while the sum of squares was used as the error function (Battiti and Massuli 1990).

The performance of ANN models was assessed with the Spearman’s rank correlation coefficient calculated between experimental and predicted data, separately for the training and verification subsets which was done automatically. The same was calculated for the best performing models where the correlation between real and predicted data for each site was examined. Additionally, the root mean squared error (RMSE) for each model was calculated. Next, sensitivity analysis was performed in order to rank input variables based on the model error calculated when a given input variable is removed from the network. The ratio of the error for the complete model to the error for a model with one variable subtracted indicated the relative importance of the variables. Statistical analyses were performed using StatSoft software Statistica 9.0 and Statistica 12.0 Trial (Lula 2000; StatSoft 2011; Tadeusiewicz and Lula 2001).

MRT models were computed using the R (v. 2.9.2) statistical environment to detect threshold values above which *Leptosphaeria* ascospore concentration significantly increased in the air of study sites using the mvpart (Multivariate Partitioning) package (De’Ath 2002). Repeated splitting of data along the axes of explanatory variables allowed forming clusters and each split was chosen to maximize the similarity within tree nodes. Two thousand multiple cross-validations were computed in order to stabilize the cross-validated relative error (CV error). The final MRT models were chosen based on the lowest value of the CV error and standard error (SE).

### Bioclimatic and vegetation spatial analyses

The Global Land Cover Share Database v. 1.0 (FAO/Land and Water Division 2014) was employed to yield a group of maps showing the distribution of (a) dominant land cover type, (b) crops, and (c) grasslands, which constitutes source areas of *Leptosphaeria* species. All maps were gridded with a resolution equal to 250 m^2^. More detailed, Corine Land Cover 2006 could not be used in this study since it does not include the relevant information for the territory of Ukraine. However, the GLC-SHARE database is a greatly improved version of the previously released Global Land Cover 2000 database (2003) since it now contains more detailed information at regional and country levels and has been harmonized using internationally accepted standards and definitions.

WorldClim—Global Climate Data (v. 1.4, release 3) was used in order to examine the bioclimatic conditions, specific for each of the studied site (Hijmans et al. 2005). This data set contains an average of real data points measured throughout a 50-year period (~ 1950–2000). The applied grid was equal to 1 km^2^. The following maps were produced using this database: (a) annual mean temperature, (b) mean temperature of warmest quarter, (c) annual precipitation, (d) precipitation of warmest quarter.

All maps were produced and analyzed using ArcGIS (ArcMap v. 10.0).

## Results

### Vegetation and bio-climate

An analysis of the GLC-SHARE data showed that the most dominant land cover class was the cropland areas in Europe, although the intensity of agriculture varied greatly between countries. All study sites were located in the vicinity of large agricultural complexes while Szczecin was also surrounded by large woodland areas. These results were investigated further, and overall croplands covered from 81 to 100% territory near Worcester and Szczecin. Lower values were found for Vinnytsya and Zaporizhia, where agriculture utilizes between 61 and 80% of the land. Similarly, grasslands covered less area in Vinnytsya and Zaporizhia (< 20%) than at Szczecin (< 40%) or Worcester (< 60%).

The bioclimatic data, made available by the FAO/Land and Water Division (2014), showed that all study sites experienced annual mean temperature within the range of 5–10 °C while the clearest differences arose during the warmest quarter of the year. The mean temperatures within a given range were as follows: the lowest was found at Worcester (10–15 °C), higher at Szczecin and Vinnytsya (15–20 °C), and the highest at Zaporizhia (20–25 °C). With regard to the annual sum of precipitation, then the lowest amount of rainfall was recorded at Zaporizhia (250–500 mm) while double this amount was observed at the other locations. During the warmest quarter of the year, Vinnytsya received up to 400 mm of rain, which was the highest value.

### Seasonality in ascospore dissemination

Although the maximum concentration was recorded in Vinnytsya in 2009, where ascospores peaked reaching a value of 1826 s m^−3^, overall the highest levels of *Leptosphaeria* ascospores were observed in Worcester (Table 2). The lowest concentrations were recorded at Zaporizhia. On average, daily concentrations were found to be 8 s m^−3^ (Zaporizhia), 79 s m^−3^ (Vinnytsya), 88 s m^−3^ (Szczecin), and 177 s m^−3^ (Worcester).

**Table 2:** Descriptive statistics of *Leptosphaeria* ascospore concentrations at study sites in Poland, the UK, and Ukraine (2006–2012)

Site	Szczecin PL				Worcester UK				Vinnytsya UA				Zaporizhia UA			
Year	2006	2007	2008	2009	2006	2007	2008	2009	2009	2010	2011	2012	2009	2010	2011	2012
Mean	75	127	72	77	135	156	237	180	65	151	51	50	23	5	2	2
Median	31	86	23	49	59	90	112	97	22	58	15	7	8	0	0	0
Minimum	0	0	2	0	0	0	2	0	0	0	0	0	0	0	0	0
Maximum	540	648	486	423	983	796	1350	1193	1826	1255	860	660	352	133	72	62
Range	540	648	484	423	983	796	1348	1193	1826	1255	860	660	352	133	72	62
25%	8	15	6	13	13	25	38	22	8	15	4	1	3	0	0	0
75%	107	191	100	118	203	224	332	284	63	171	51	51	20	0	0	0
Standard deviation	97	130	98	82	171	172	286	214	153	239	103	100	47	18	9	9
Variance	9389	16,906	9529	6712	29,201	29,429	81,915	45,835	23,386	57,306	10,544	9986	2215	317	74	77

Results of Spearman’s rank test for correlation between ascospore concentration and meteorological measurements are given in Table 3. In Szczecin, Worcester, and Vinnytsya, all correlations between *Leptosphaeria* ascospore concentration and meteorological parameters exceeded the level of statistical significance (*p* < 0.05), except for average wind speed at Szczecin. At Zaporizhia, this was true for association with dew point temperature (DPT) and average wind speed (Table 3). The strength of statistically significant correlations varied greatly while the highest were found with DPT at Szczecin (*r*_*s*_ = 0.61) and Worcester (*r*_*s*_ = 0.54).

**Table 3:** Results of Spearman’s rank test examining the association between *Leptosphaeria* ascospore concentration and meteorological parameters at study sites in Poland, the UK, and Ukraine for a 4-year period (1 Mar–31 Oct)

Site	Szczecin^a^	Worcester^a^	Vinnytsya^b^	Zaporizhia^b^
AVAT	*0.478*	*0.340*	*0.414*	0.031
MAAT	*0.414*	*0.340*	*0.377*	0.030
MIAT	*0.528*	*0.340*	*0.468*	0.028
DPT	*0.606*	*0.544*	*0.501*	*0.085*
RH	*0.095*	*0.456*	*0.098*	− 0.033
PREC	*0.196*	*0.449*	*0.149*	0.045
AVWS	− 0.004	*− 0.097*	*− 0.083*	**−** *0.078*

Statistically significant correlations at *p* < 0.05 were highlighted in italics

*AVAT* average air temperature, *MAAT* maximum air temperature, *MIAT* minimum air temperature, *DPT* dew point temperature, *RH* relative humidity, *PREC* precipitation, *AVWS* average wind speed

^a^2006-2009

^b^2009-2012

### Multivariate regression trees

An analysis of *Leptosphaeria* ascospore presence in the air of studied sites using the multivariate regression trees indicated the importance of DTP in models computed for Szczecin, Worcester, Vinnytsya, and Zaporizhia as well as the joint models for Szczecin+Worcester and Vinnytsya+Zaporizhia. For these models, DPT was found to either the first or second most important parameter for predicting ascospore release with precipitation and average wind speed being the other highly ranked parameters (Supplementary materials, Fig. S1-A – S1-F*).* DPT reached a value of 8.25 and 8.22 °C at the first split (Szczecin and Szczecin+Worcester, respectively). At secondary splits, it was 4.80 and 6.79 °C for Szczecin and Szczecin+Worcester, while for Worcester the values of 6.79 and 10.42 °C were obtained. The amount of recorded precipitation was, however, found to be more influential at Zaporizhia (2.75 mm) and Worcester (0.90 mm) at first tree splits regardless of the presence or absence of DPT as an explanatory factor in these models. In case of models obtained for the Vinnytsya site, the primary explanatory variable was DPT, at the level of 15.02 °C, followed by minimum air temperature (17.85 °C). The joint model for Vinnytsya and Zaporizhia showed the influence of average wind speed, followed by DPT. Various impacts of other, less important parameters, such as relative humidity at Szczecin (68.69%), minimum air temperature at Zaporizhia (13.25 °C), average wind speed at Worcester (9–13 m s^−1^), and minimum air temperature for Vinnytsya+Zaporizhia (9.75 °C) were also revealed.

### Artificial neural networks

#### Ascospore prediction for single sites

**Fig. 3:**
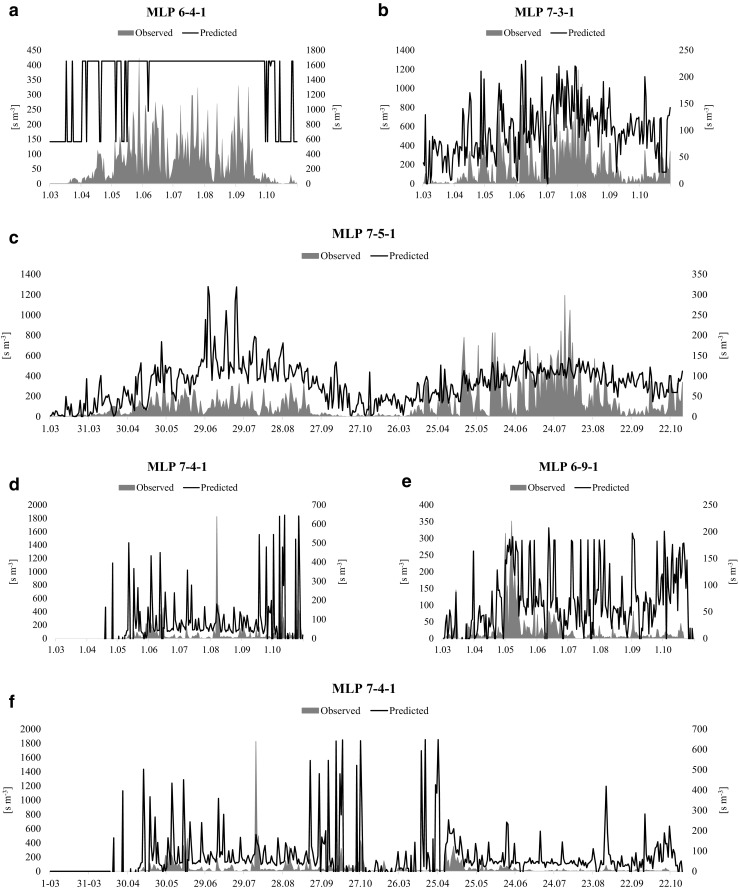
Overall performance of the best forecasting models (highest correlation coefficient values between observed and predicted spore concentration calculated for all the subsets together) obtained for **a** Szczecin (MLP 6-4-1), *r*_s_ = 0.608 **b** Worcester (MLP 7-3-1), *r*_s_ = 0.631 **c** Szczecin+Worcester (MLP 7-5-1), *r*_s_ = 0.564 **d** Vinnytsya (MLP 7-4-1), *r*_s_ = 0.305 **e** Zaporizhia (MLP 6-9-1), *r*_s_ = 0.430 **f** Vinnytsya+Zaporizhia (MLP 7-4-1), *r*_s_ = 0.327

The ANN model for prediction of *Leptosphaeria* concentrations, computed using Worcester data (MLP 6-4-1), was found to be the best predicting model for Szczecin. This model was a multi-layer perceptron with six input neurons, four hidden neurons and one output neuron. The performance of this network was good although the ascospore concentrations were overestimated (Table 4, Fig. 3a). The Spearman’s rank correlation coefficients between the observed and predicted values were at the level of 0.72 and 0.71 for the training and validation subsets, respectively. The final model was trained with 714 epochs of the Broyden-Fletcher-Goldfarb-Shanno algorithm. Hidden neurons had logistic activation functions while the output neuron had an exponential activation function. Sensitivity analysis revealed that all the variables contributed to the model. The most important variables turned out to be all the temperature parameters, in descending order—maximum, mean, and minimum. Other examined variables also contributed to the model, but less significantly. Comparison of the experimental and predicted values of *Leptosphaeria* spore concentrations showed good performance (Fig. 3a). The Spearman’s correlation coefficient between the real data and model prediction was at the level of *r*_*s*_ = 0.61 (*p* < 0.001).

**Table 4:** Results tests examining the association between observed *Leptosphaeria* ascospore concentration and predicted concentrations at study sites in Poland, United Kingdom and Ukraine using data collected in 2009 (1 Mar–31 Oct). The best performing models were presented for each tested combination

Model	Data set used for testing					
	Szczecin	Worcester	Szczecin+Worcester	Vinnytsya	Zaporizhia	Vinnytsya+Zaporizhia
(−) Szczecin		*R* = 0.615 RMSE = 47.55		*R* = −0.181 RMSE = 510.20	*R* = 0.390 RMSE = 210.45	*R* = 0.299 RMSE = 315.33
(−) Worcester	*R* = 0.608 RMSE = 56.12			*Not found*	*R* = 0.209 RMSE = 338.95	*R* = −0.197 RMSE = 503.12
(−) Szczecin+Worcester				*R* = 0.201 RMSE = 349.50	*R* = 0.430 RMSE = 215.45	*R* = −0.239 RMSE = 362.45
(−) Vinnytsya	*R* = 0.589 RMSE = 98.12	*R* = 0.368 RMSE = 231.12	*R* = 0.453 RMSE = 201.13		*R* = 0.212 RMSE = 338.34	
(−) Zaporizhia	*R* = 0.563 RMSE = 153.12	*R* = 0.461 RMSE = 192.18	*R* = 0.470 RMSE = 189.65	*R* = −0.241 RMSE = 321.15		
(−) Vinnytsya+Zaporizhia	*R* = 0.262 RMSE = 366.6	*R* = −0.331 RMSE = 251.54	*R* = −0.310 RMSE = 243.11			
(+) Szczecin		*R* = 0.631 RMSE = 35.91		*R* = 0.305 RMSE = 261.78	*R* = 0.374 RMSE = 261.82	*R* = 0.327 RMSE = 259.21
(+) Worcester	*R* = 0.261 RMSE = 345.11			*R* = −0.278 RMSE = 320.11	*R* = 0.213 RMSE = 329.66	*R* = −0.223 RMSE = 358.43
(+) Szczecin+Worcester				R = 0.212 RMSE = 333.56	R = 0.368 RMSE =220. 21	*R* = 0.302 RMSE = 268.62
(+) Vinnytsya	*R* = 0.556 RMSE = 117.18	*R* = 0.617 RMSE = 46.23	*R* = 0.564 RMSE = 101.43		*R* = 0.226 RMSE = 245.67	
(+) Zaporizhia	*R* = 0.588 RMSE = 97.15	R = 0.363 RMSE = 220.87	*R* = 0.418 RMSE = 201.33	*R* = −0.208 RMSE = 344.22		
(+) Vinnytsya+Zaporizhia	*R* = 0.286 RMSE = 333.25	*R* = 0.363 RMSE = 219.33	*R* = 0.274 RMSE = 320.73			

All presented models were statistically significant (*p* < 0.05). Models containing dew point temperature were marked (+) and models without this variable were marked (−)

*Not found* no statistically significant forecasting model was found, *R* Spearman’s rank correlation coefficient, *RMSE* root mean squared error

The ANN model designed for Szczecin (MLP 7-3-1) provided the best model for *Leptosphaeria* ascospore concentrations in Worcester. This network was built of seven input neurons, three hidden neurons, and one output neuron. The Spearman’s rank correlation coefficient between the experimental and estimated spore concentrations was at the level of 0.61 and 0.53 for the training and validation subsets, respectively. The selected model was trained with 10,000 epochs of the BFGS algorithm. The sum of squares was used as the error function. Both hidden and output neurons had an exponential activation function. The obtained ANN model revealed a good fit to the experimental data in terms of high-low changes but not in terms of the actual maximum concentrations (Fig. 3b). Sensitivity analysis demonstrated that all the explanatory variables were almost equally important to the model, with DPT ranked the highest followed by mean air temperature and relative humidity. The Spearman’s correlation coefficient between the real data and model prediction was *r*_*s*_ = 0.63 (*p* < 0.001).

The best performing model for Vinnytsya was the one obtained using Szczecin data (MPL 7-4-1) (Table 4). This ANN network was a multi-layer perceptron comprised of seven input neurons, four hidden neurons and one output neuron (MLP 7-4-1). The Spearman’s rank correlation coefficients between the real and calculated values were at the level of 0.63 and 0.53 for the training and validation subsets, respectively. The model was trained with 716 epochs of the BFGS algorithm. The sum of squares was applied as the error function. Hidden neurons had logistic activation functions while the output neuron had a sine activation function. Sensitivity analysis revealed that all variables contributed significantly to the model (scores > 1). However, this was the poorest performing model (Fig. 3d), which was confirmed by the Spearman’s correlation coefficient between the real data and model output (*r*_*s*_ = 0.31, *p* < 0.001).

Finally, ascospore concentrations for Zaporizhia were the most accurately forecasted with the aid of an ANN model (MLP 6-9-1) computed using combined Szczecin and Worcester data (Table 4). This model was a multi-layer perceptron with six input neurons, nine hidden neurons, and one output neuron. The Spearman’s rank correlation coefficients between the experimental and calculated values were at the level of 0.73 and 0.69 for the training and validation subsets, respectively. The model was trained with 10,000 epochs of the Broyden-Fletcher-Goldfarb-Shanno algorithm. The sum of squares was applied as the error function. Hidden neurons had tangent activation functions while the output neuron had a linear activation function. Sensitivity analysis revealed that all the variables contributed to the model (scores > 1); it was found that most important were mean, minimum, and maximum temperature. Model performance was good from March to July, and then suddenly decreased resulting in substantial overestimation of ascospore concentrations (Fig. 3e). Thus, the Spearman’s correlation coefficient between the real data and model forecast was equal to *r*_*s*_ = 0.43 (*p* < 0.001).

#### Two-site combined models

Two combined models were produced using joint data, i.e., Szczecin+Worcester (West Europe) and Vinnytsya+Zaporizhia (East Europe). Overall, the performance of two-site models was lower than those for single sites (Table 4). The best performing model for Szczecin+Worcester data was the multi-layer perceptron, which consisted of seven input neurons, five hidden neurons, and one output neuron (MLP 7-5-1) produced for Vinnytsya (Table 4). The Spearman’s rank correlation coefficients between the experimental and calculated values were at the level of 0.47 and 0.41 for the training and validation subsets, respectively. The model was trained with 10,000 epochs of the Broyden-Fletcher-Goldfarb-Shanno algorithm. The sum of squares was applied as the error function. Hidden neurons had exponential activation functions while the output neuron had an exponential activation function. Sensitivity analysis revealed that all the variables contributed to the model (scores > 1) with the most important being maximum temperature, followed by mean and minimum temperature. Model performance was quite low (Fig. 3c); however, the Spearman’s correlation coefficient between the real data and model forecast was equal to *r*_*s*_ = 0.56 (*p* < 0.001). The model overestimated the spore concentrations but accurately predicted their fluctuations.

The multi-layer perceptron, which consisted of seven input neurons, four hidden neurons, and one output neuron (MLP 7-4-1) produced for Szczecin, was the best performing model for Vinnytsya+Zaporizhia data (Table 4). The Spearman’s rank correlation coefficients between the experimental and calculated values were at the level of 0.63 and 0.53 for the training and validation subsets, respectively. The model was trained with 716 epochs of the Broyden-Fletcher-Goldfarb-Shanno algorithm. The sum of squares was applied as the error function. Hidden neurons had logistic activation functions while the output neuron had a sine activation function. Sensitivity analysis revealed that all the variables contributed towards model performance. The highest scoring parameter was DPT, followed by mean and maximum temperature. Model performance was very poor (Fig. 3f), as the Spearman’s correlation coefficient between the real data and model forecast was equal to *r*_*s*_ = 0.33 (*p* < 0.001).

## Discussion

In this study, we proposed an application of artificial neural modeling in order to predict the concentration of allergenic and phytopathogenic *Leptosphaeria* spores in the air of urban areas. Out of 120 selected models, the best performing model was an MLP 7-3-1 neural model using 4 years of data collected in Worcester and examined using an 8-month period in 2009. The model was most suitable for both Worcester in the UK and for Szczecin in Poland. The performance of this model was relatively good, as it correctly predicted the spore concentration in 63% of days. Artificial neural networks computed for Szczecin and tested with Worcester data showed a better performance than models produced for Worcester and cross-checked with Szczecin data. However, a better agreement was found between the models computed for Szczecin and Worcester than the models derived and reciprocally tested for the two cities in Ukraine. Seemingly, the overall distribution of *Leptosphaeria* ascospores in the air of Vinnytsya must be of a greater variation (considerable fluctuations and higher concentrations in the data over the sampling period) compared with Zaporizhia and, hence, more difficult to predict.

Ascospores of some *Leptosphaeria* species are produced and released mainly in autumn (West and Fitt 2005; Kaczmarek and Jedryczka 2011). In a series of studies carried out by Kaczmarek et al. (2009, 2012, 2014) climatic differences between the oilseed rape-cultivating ecological zones significantly affected biological processes influencing the infection cycle of two pathogenic *Leptosphaeria* species causing stem canker of oilseed rape.

A reasonably uniform pattern in the distribution of spores is expected when spore inoculum source is located in the vicinity of the air sampler and it is independent of the weather. Jedryczka et al. (2013) and Kaczmarek et al. (2014) postulated that the irregularity and differences in the dynamic of sporulation of *L. maculans* and *L. biglobosa* required constant monitoring of air samples for the benefit of farmers, whose crops may be strongly affected by the disease originating from airborne inoculum.

Results of studies of *Leptosphaeria* conducted in Szczecin (Grinn-Gofroń et al. 2016) showed that spores had a heterogeneous origin and occasionally were blown in from crops located up to 400 km away. Although events of long distance transport described in that study occurred at the beginning of July, such events could well occur throughout the year. With regard to Worcester, the performance of models could stem from a possible shorter distance between the air sampler and *Leptosphaeria* host plants and stable spectrum of winds due to local topography (Sadyś et al. 2014).

Sensitivity analysis examined the impact of weather parameters on *Leptosphaeria* ascospores presence and concentration. Models, which were computed based on continuous 4-year observations uniformly indicated mostly maximum temperature, mean, and minimum temperature, although the order of importance varied between models. Out of the top six models presented in this paper, four of them included DPT as an explanatory variable. The only two exceptions were the Worcester (MLP 6-4-1) and Szczecin+Worcester (MLP 6-9-1) models, which yielded the highest performance without including DPT as an input variable. Out of the four locations studied, Worcester was the wettest site, where DPT was not found as a good explanatory variable and thus it was removed from the forecast model. In spite of this, DPT was still indicated as significant by the sensitivity analysis. This calculation showed DPT in Worcester was still the key factor influencing ascospore concentrations, which was then confirmed by the Spearman’s rank test and MRT analysis. The same model is also influenced by the changes in relative humidity. Our results were, therefore, similar to those reported elsewhere, where Spearman’s rank test confirmed positive correlation between spore concentration and rainfall (Szczecin, Worcester, Vinnytsya), relative humidity (Szczecin, Worcester, Vinnytsya), DPT (Szczecin, Worcester, Zaporizhia), which trigger ascospores release when matured (Hernández Trejo et al. 2012; Sadyś et al. 2015; Salam et al. 2007). The impact of relative humidity, as well as maximum wind speed on the presence of *Leptosphaeria* ascospores, was also suggested by Grinn-Gofroń and Bosiacka (2015), who analyzed these relationships with the aid of Canonical Correspondence Analysis.

Salam et al. (2003) produced a regression model based on measurements of daily mean temperature and rainfall in order to predict maturation of fungus pseudothecia and ascospore discharge. The model generated functioned very well; it explained from 66 to 93% of the variability in spore fluctuations, although the performance of the model was a subject of change with respect to the sampling site and season. Dawidziuk et al. (2012) computed a stepwise regression model, based solely on the analysis of precipitation. They found that 77% of the *Leptosphaeria* distribution depended on the amount of rainfall recorded during the first decade of July together with a cumulative amount of rainfall during the entire month of July. This resulted in very good performance of the model, which showed a strong correlation (*r* = 0.88) between observed and predicted spore concentration. This correlation was superior compared to this obtained for our best performing model (*r*_*s*_ = 0.63). Papastamati et al. (2004) proposed a physical model, where they examined the impact of duration of leaf wetness during the rosette stage of canola plants; this enabled them to predict 81–97% of blackleg infections in a season. Although the model worked very well, it required an interaction with a second model that predicted production of ascospores and only then could it be applied to forecasting.

As reviewed by Després et al. (2012) numerous approaches have been used for modeling the transport of different biological particles, including Gaussian plume models, Lagrangian stochastic models, as well as models concerning the effect of climate and transport at regional or global scale. Most of these studies focused on the spread of microbes responsible for the diseases of humans, animals and plants, and most of published models obviously performed well. However, one has to keep in mind that good performance of any model is often limited to the location of sampling (Papastamati et al. 2004). Thus, testing the model using a data set collected elsewhere may not result in equally good performance. This finding concerned not only the geographical coordinate of the sampling site, but also the height of the sampler; in spore monitoring of *Ganoderma* ANN models worked well for samples collected 18 m a.g.l., but they were inaccurate or wrong for spore data originating from the samplers located on the ground (Jedryczka et al. 2015). This brings into question the use of such forecasting models for warning of farmers, foresters as well as patients with inhalatory spore-related allergies.

## Conclusion

This study, successfully applies artificial neural modeling to predict the concentration of allergenic *Leptosphaeria* spores in the air of urban areas for the first time for this genus. The work demonstrated that local micro-climate plays a key role in formation of teleomorphs and the release of ascospores of *Leptosphaeria* spp. Artificial neural network computation did not produce a universal forecast model that would perform equally well at sites greatly differing in weather parameters, but the models produced for individual sites were satisfactory. Both the multivariate regression tree analysis and Spearman’s rank test showed a great impact of DPT; MRT analyses indicated its importance in five out of six cases and in three cases ranked it as a fundamental weather parameter. The findings are in agreement with the current knowledge on the behavior of *Leptosphaeria* spp., as both humidity and temperatures were previously indicated as important parameters in pseudothecia maturation and the release of ascospores. The elucidation of the crucial role of DPT for the formation of inoculum, which is allergenic and subsequently damaging to wild and cultivated agricultural plants, will facilitate the ongoing search for good forecasting models for *Leptosphaeria* spp.

## Electronic supplementary material

ESM 1 (DOC 63 kb)
