# Supplementary material for: Dew point temperature affects ascospore release of allergenic genus Leptosphaeria
Source: Int J Biometeorol. 2018 Jan 27;62(6):979–90. doi: 10.1007/s00484-018-1500-z (PMC5966494; doi:10.1007/s00484-018-1500-z)
Supplement: Supplementary file 1 — (DOC 63 kb) [file 484_2018_1500_MOESM1_ESM.doc]

***Supplementary materials to the article***

**Dew Point Temperature affects ascospore release of allergenic genus *Leptosphaeria***

Magdalena Sadyś ^1,2^, Joanna Kaczmarek ^3^, Agnieszka Grinn-Gofron ^4^, Victoria Rodinkova ^5^, Alex Prikhodko ^6^, Elena Bilous ^5^, Agnieszka Strzelczak ^7^, Robert J. Herbert ^2^, Malgorzata Jedryczka ^3#^

^1^ Rothamsted Research, West Common, Harpenden, AL5 2JQ, UK

^2^ Institute of Science and the Environment, University of Worcester, Henwick Grove, Worcester, WR2 6AJ, UK

^3^ Institute of Plant Genetics, Polish Academy of Sciences, Strzeszynska 34, 60-479 Poznan, Poland

^4^ Department of Plant Taxonomy and Phytogeography, University of Szczecin, Waska 13, 71-415 Szczecin, Poland

^5^ Vinnytsya National Pirogov Memorial Medical University, Vinnytsya, Ukraine

^6^ Zaporizhia State Medical University, Zaporizhia, Ukraine

^7^ West Pomeranian University of Technology, Faculty of Food Sciences and Fisheries, Department of Food Process Engineering, Papieza Pawla VI 3, 71-459 Szczecin, Poland

^#^ Corresponding author: mjed@igr.poznan.pl

Fig. S1-A Multivariate regression tree models produced for Szczecin; *top* – model including dew point temperature, *down* – model computed without dew point temperature. Both models were produced upon 4-year data (1 Mar - 31 Oct 2006-2009).

Fig. S1-B Multivariate regression tree models produced for Worcester; *top* – model including dew point temperature, *down* – model computed without dew point temperature. Both models were produced upon 4-year data (1 Mar - 31 Oct 2006-2009).

Fig. S1-C Multivariate regression tree models produced jointly for Szczecin+Worcester; *top* – model including dew point temperature, *down* – model computed without dew point temperature. Both models were produced upon 4-year data (1 Mar - 31 Oct 2006-2009).

Fig. S1-D Multivariate regression tree models produced for Vinnytsya; *top* – model including dew point temperature, *down* – model computed without dew point temperature. Both models were produced upon 4-year data (1 Mar - 31 Oct 2009-2012).

Fig. S1-E Multivariate regression tree models produced for Zaporizhia; *top* – model including dew point temperature, *down* – model computed without dew point temperature. Both models were produced upon 4-year data (1 Mar - 31 Oct 2009-2012).

Fig. S1-F Multivariate regression tree models produced for Vinnytsya+Zaporizhia; *top* – model including dew point temperature, *down* – model computed without dew point temperature. Both models were produced upon 4-year data (1 Mar - 31 Oct 2009-2012).
